# Cancer prevalence among flight attendants compared to the general population

**DOI:** 10.1186/s12940-018-0396-8

**Published:** 2018-06-26

**Authors:** Eileen McNeely, Irina Mordukhovich, Steven Staffa, Samuel Tideman, Sara Gale, Brent Coull

**Affiliations:** 1 Department of Environmental Health, Harvard T.H. Chan School of Public Health, Landmark Center, 401 Park Dr, Boston, MA 02215 USA; 2 Department of Biostatistics, Harvard T.H. Chan School of Public Health, Boston, MA USA

**Keywords:** Flight attendants, Occupational epidemiology, Cancer, Environmental health

## Abstract

**Background:**

Flight attendants are an understudied occupational group, despite undergoing a wide range of adverse job-related exposures, including to known carcinogens. In our study, we aimed to characterize the prevalence of cancer diagnoses among U.S. cabin crew relative to the general population.

**Methods:**

In 2014–2015, we surveyed participants of the Harvard Flight Attendant Health Study. We compared the prevalence of their self-reported cancer diagnoses to a contemporaneous cohort in the National Health and Nutrition Examination Survey (NHANES 2013–2014) using age-weighted standardized prevalence ratios (SPRs). We also analyzed associations between job tenure and the prevalence of selected cancers, using logistic regression and adjusting for potential confounders.

**Results:**

Compared to NHANES participants with a similar socioeconomic status (*n* = 2729), flight attendants (*n* = 5366) had a higher prevalence of every cancer we examined, especially breast cancer, melanoma, and non-melanoma skin cancer among females. SPR for these conditions were 1.51 (95% CI: 1.02, 2.24), 2.27 (95% CI: 1.27, 4.06), and 4.09 (95% CI: 2.70, 6.20), respectively. Job tenure was positively related to non-melanoma skin cancer among females, with borderline associations for melanoma and non-melanoma skin cancers among males. Consistent with previous studies, we observed associations between job tenure and breast cancer among women who had three or more children.

**Conclusions:**

We observed higher rates of specific cancers in flight attendants compared the general population, some of which were related to job tenure. Our results should be interpreted in light of self-reported health information and a cross-sectional study design. Future longitudinal studies should evaluate associations between specific exposures and cancers among cabin crew.

**Electronic supplementary material:**

The online version of this article (10.1186/s12940-018-0396-8) contains supplementary material, which is available to authorized users.

## Background

Flight attendants are a highly understudied occupational cohort that is consistently exposed to several known and probable carcinogens in the cabin environment [1]. These include cosmic ionizing radiation at flight altitude, Circadian rhythm disruption due to night shift work, irregular schedules and frequently crossing time zones, and poor cabin air quality from a number of sources [2–4]. Many flight attendants working today were also exposed to high levels of secondhand tobacco smoke before in-flight smoking bans were implemented [5]. The long-term health effects of this mix of occupational exposures, including with regard to cancers which develop over the course of many years, have not been well characterized.

Until 2014, flight attendants were excluded from Occupational Safety and Health Administration protections typically granted to U.S. workers, and only limited protections (such as with regard to blood-borne pathogens) were implemented in 2014. Flight attendants’ exposure to ionizing radiation is still not monitored or regulated in any way, despite the fact that cabin crew are exposed to the largest average annual effective dose relative to all other U.S. radiation workers [6]. Studies regarding cancer risk or prevalence among flight attendants are relatively sparse and of varying quality [2]. Results from these studies have been mixed, but overall point towards associations between in-flight exposures or job tenure as a flight attendant and increased rates of breast and skin cancers, as well as aggregated cancers at all sites [7–11].

To address gaps in the literature, we launched the Harvard Flight Attendant Health Study (FAHS) in 2007 [12]. In the first wave of our study, we compared cancer rates of cabin crew relative to the general population, and evaluated associations between job tenure as a proxy for occupational exposures and broad groupings of cancer types. This study reported elevated rates of female reproductive cancers among cabin crew, as well as an association between job tenure and the prevalence of aggregated skin cancers, but the survey did not query participants about individual cancer diagnoses [12]. We have since completed the second wave of the FAHS in 2014–2015, in which we also reported associations between flight attendant work and the prevalence of cancer at all sites and aggregated reproductive cancers [1]. We now aim to characterize the prevalence of a wide range of specific cancer diagnoses among this occupational group relative to the U.S. general population and in relation to job tenure and parity. We hypothesized that we would observe associations between work as a flight attendant and prevalence of reproductive, melanoma, and non-melanoma skin cancers.

## Methods

### Study population

Our participants were enrolled in the second wave of the FAHS, an ongoing study of flight attendant health which was established in 2007 and originally enrolled 4011 flight attendants [12]. For the 2014–2015 wave of the FAHS reported in this manuscript, we recruited both new and returning flight attendants to participate through several channels, including a hard copy survey mailed to the 2007 participants and distributed at airport terminals between December 2014 and June 2015, and an online survey launched in December 2014 [1]. We supplemented our survey reach with in-person recruitment at five large airport hubs in the U.S. Our recruitment campaign also included announcements about the study from local unions and through social media. Survey participants were eligible to enter a lottery to win an iPad or Apple watch over an 18-month period.

Any current or former U.S. flight attendant was eligible to participate in the FAHS (91% of the participants in the current study were currently employed as flight attendants, and 9% were former flight attendants). While Wave 1 of the FAHS restricted participants to flight attendants employed by two U.S. airlines, Wave 2 was open to any U.S. flight attendant. Hence, participants in the current study worked for a wide range of airlines, flying both domestically and internationally. We collected 1642 surveys from returning participants, which represents a 40% response rate from the original cohort with still-valid addresses. In total, the 2014–2015 FAHS cohort enrolled 5366 U.S. flight attendants with information on age and gender. These variables were among the last questions to be asked in the online questionnaire and are thus indicators of survey completeness. Our study was approved the Harvard T.H. Chan School of Public Health Institutional Review Board, and all participants provided their written informed consent.

### Survey

Our survey instrument included validated questions about self-reported health outcomes and symptomology, work experiences, and personal characteristics [1, 11], taken from established surveys such as the Job Content Questionnaire and the National Health and Nutrition Examination Survey (NHANES) [13, 14]. We note that dates of cancer diagnoses were not recorded in the FAHS questionnaire. Participants were also asked to provide aviation employment history, including airlines, primary hubs, and dates of employment and leave.

### Comparison to NHANES

We compared the prevalence of cancers reported in the FAHS to equivalent information collected from a nationally representative sample from the NHANES during the years 2013–2014 [14]. The NHANES is administered by the U.S. Centers for Disease Control and Prevention, and collects demographic, health, dietary, and biomarker data from approximately 5000 U.S. residents each year. All cancers had binary answer choices for prevalence (ever diagnosed: yes/no) in the NHANES questionnaire. We weighted the NHANES data by their two-year sample weights, primary sampling units, and strata based on published analytic guidelines [15]. We restricted respondents to currently employed adults with a family income to poverty ratio of 1 or greater and at least a high school education in order to better match the demographic and socioeconomic characteristics of our study population.

### Statistical analyses

We compared the prevalence of self-reported cancers in the NHANES and FAHS using the Standardized Prevalence Ratio (SPR), an indirect method of standardization which compares observed and expected prevalence given rates in the reference study population, which in our case was the NHANES [16]. The SPR was weighted by age category (18–39, 40–59, and 60+ years) and analyzed separately by gender. We conducted these analyses for cancers diagnosed among at least 20 FAHS participants for a given gender. To further increase the comparability of the two study populations, we conducted a sensitivity analysis restricted to non-Hispanic white participants, who comprised 75% of our cohort and 43% of the NHANES population. We also conducted a secondary analysis evaluating the age-adjusted comparative prevalence of cancers for flight attendants exposed to high levels of historical occupational secondhand smoke prior to 1988, standardized to the subset of NHANES participants ages 45 or older. Occupational smoking exposure was based on reported work histories – those working as flight attendants prior to 1988 were considered to be in the highly exposed subgroup.

We analyzed gender-stratified associations between net job tenure (total time working as a flight attendant minus any leave) and self-reported cancers using logistic regression and adjusting for potential confounders: age (continuous), current and past smoking status (yes/no for both), current overweight status based on body mass index (25+ vs. < 25 kg/m^2^), and educational attainment (high school, some college/trade certificate, college degree or higher); breast cancer models were further adjusted for number of live births. Tenure was meant to serve as a proxy for the duration of occupational exposures [17]. We also conducted sensitivity analyses further adjusting multivariate tenure-cancer models for usual alcohol intake (none, 1–3 servings per month, 1–6 servings per week, 1 or more servings per day), as alcohol consumption is a risk factor for breast cancer [18] and may be a risk factor for melanoma and non-melanoma skin cancers as well [19].

We examined cancer prevalence in relation to total net tenure as well as tenure prior to age 40 and age 45 (restricted to participants aged over 40 and 45, respectively, in order to standardize exposure opportunity). For breast cancer, we evaluated these associations both overall and stratified by parity (nulliparous, 1–2, 3+). Parity categories were chosen based on previous studies of breast cancer among cabin crew [20, 21]. We chose to examine tenure at younger ages in order to approximate exposures during the reproductive years for hormone responsive cancers, and because of evidence that ionizing radiation exposure is most relevant to cancer risk at earlier ages [22, 23]. Finally, we conducted a sensitivity analysis restricting SPRs and tenure analyses to the 91% of our cohort who were currently employed as flight attendants to better match the NHANES comparison population. Analyses were completed using STATA statistical software, version 14 (StataCorp, College Station, TX).

## Results

We report characteristics of both FAHS and NHANES participants in Additional file 1: Table S1. Wave 2 FAHS participants presented with a mean age of 52 years (15% were between ages 18–39, 54% were between ages 40–59, and 32% were over age 60) and an average net job tenure of 20 years. Over 80% of our cohort was female, as expected in this feminized occupation, and 8% reported being current smokers. Slightly over 15% of our participants reported ever being diagnosed with any cancer. NHANES participants’ ages differed from FAHS participants, with 31% between ages 18 and 39, 50% between ages 40 and 59, and 20% over age 60. Mean number of live births also differed between the females in the two study populations (0.8 for FAHS vs. 2.1 for NHANES), despite NHANES having a higher percentage of women still in their childbearing years than the FAHS. The NHANES cohort was 54% female, and 16% reported being current smokers (Additional file 1: Table S1).

We report SPRs comparing the prevalence of cancer diagnoses in the FAHS and the NHANES in Table 1. We report a higher prevalence of every cancer outcome we examined among cabin crew relative to the general population, including breast, uterine, cervical, gastrointestinal, thyroid, melanoma, and non-melanoma skin cancers. The SPRs for breast, melanoma and non-melanoma skin cancers among females were 1.51 (95% CI: 1.02–2.24), 2.27 (95% CI: 1.27–4.06), and 4.09 (95% CI: 2.70–6.20), respectively, with slightly higher SPRs among those with high occupational secondhand smoke exposure (Additional file 2: Table S2). The SPR for melanoma and non-melanoma skin cancers were modestly elevated among males overall (SPR = 1.47, 95% CI: 0.72–3.01, and SPR = 1.11, 95% CI:0.78–1.59, respectively), and were considerably higher (though less precise) among men exposed to high levels of occupational secondhand smoke (SPR = 3.80, 95% CI: 1.67–8.65 and SPR = 2.43, 95% CI: 1.53–3.87) (Additional file 2: Table S2). All reported SPRs were similar when restricting the NHANES and FAHS study populations to non-Hispanic white participants (data not shown).

**Table 1:** Comparative age-adjusted cancer prevalence in the FAHS and NHANES

Cancers	Gender	FAHS count, unweighted	NHANES count, unweighted	Prevalence FAHS, %	Prevalence, NHANES, %	SPR (95% CI)
Breast Cancer	Female	195	29	3.4	2.3	1.51 (1.02–2.24)
Uterine Cancer	Female	27	2	0.51	0.13	3.83 (0.81–18.19)
Cervical Cancer	Female	48	9	1.0	0.70	1.48 (0.73–3.00)
Gastrointestinal Cancer^a^	Female	26	4	0.47	0.27	1.73 (0.55–5.39)
Melanoma	Female	109	13	2.2	0.98	2.27 (1.27–4.06)
	Male	24	10	1.2	0.69	1.47 (0.72–3.01)
Non-Melanoma Skin Cancer	Female	418	23	7.4	1.8	4.09 (2.70–6.20)
	Male	65	42	3.2	2.9	1.11 (0.78–1.59)
Thyroid Cancer	Female	33	7	0.67	0.56	1.19 (0.53–2.66)

*CI* confidence interval, *FAHS* flight attendant health study, *NHANES* National Health and Nutritional Examination Survey; SPR: standardized prevalence ratio

^a^Includes colon, stomach, esophageal, liver, and pancreatic cancers

We also found associations between each five-year increase in net job tenure as a flight attendant and non-melanoma skin cancer among females (OR = 1.07, 95% CI: 1.01, 1.13), with borderline associations for melanoma and non-melanoma skin cancers among males (OR = 1.23, 95% CI: 0.94, 1.61 and OR = 1.17, 95% CI: 0.99, 1.38, respectively) (Table 2). Overall job tenure was not related to breast cancer, thyroid cancer, or melanoma among females. Adjusting for alcohol intake as a sensitivity analysis in our job tenure-cancer prevalence models altered associations somewhat for non-melanoma skin cancer among men (OR = 1.10 vs. 1.17) and for breast cancer (OR = 1.00 vs. 0.99), but not in relation to a 10% change in estimate criterion for determining confounding; adjusting for alcohol intake did not affect results for melanoma or non-melanoma skin cancer among women (data not shown). Sample size is lower for the tenure analysis than for the SPR calculations because of missing data regarding tenure and model covariates; missingness for these variables is reported in Additional file 1: Table S1. Similarly, restricting both the tenure analyses and the SPR calculations reported above to currently employed flight attendants (91% of the cohort) did not meaningfully alter the results, nor did restricting any of the tenure analyses to participants over the age of 40 or 45 (data not shown). With the possible exception of breast cancer, associations with most cancers were not meaningfully changed by restricting the exposure of interest to job tenure prior to age 40 or 45 (Table 3 for breast cancer; other data not shown). Finally, we found evidence of a positive association between job tenure as a flight attendant and breast cancer among those with 3 or more children, and associations were strongest when combining these parity subgroups with the job tenure measure prior to age 45. We also found some evidence of a tenure-breast cancer association among nulliparous flight attendants, though the associated confidence intervals were less precise and included the null. For example, associations between tenure prior age 45 and breast cancer risk were OR = 1.44 (95% CI: 0.83, 2.49) among nulliparous women, OR = 0.95 (95% CI: 0.78, 1.17) among women with 1 or 2 children, and OR = 1.39 (95% CI: 1.06, 1.81) among women with 3 more children (Table 3).

**Table 2:** Associations between five-year job tenure and the prevalence of specific cancer diagnoses among flight attendants^a^

Condition	Gender	N Cases	N Non-Cases	Odds Ratio (95% CI)
Breast Cancer	Female	108	2620	0.99 (0.91, 1.10)
Melanoma	Female	94	3626	1.01 (0.91, 1.13)
	Male	22	861	1.23 (0.94, 1.61)
Non-Melanoma Skin Cancer	Female	372	3348	1.07 (1.01, 1.13)
	Male	56	827	1.17 (0.99, 1.38)
Thyroid Cancer	Female	26	3694	1.00 (0.79, 1.26)

*CI* confidence interval

^a^All models are adjusted for age, overweight, education, and smoking history; the breast cancer model is further adjusted for parity

**Table 3:** Associations between five-year job tenure prior to age 45, parity and prevalence of breast cancer among female flight attendants^a^

	N Cases	N Non-Cases	Odds Ratio (95% CI)
Lifetime job tenure	108	2620	0.99 (0.91, 1.10)
Job tenure prior to age 45)^b^	90	1151	1.09 (0.91, 1.29)
Parity (lifetime job tenure)			
Nulliparous	26	1020	1.27 (0.94, 1.71)
1 to 2	68	1376	0.95 (0.84, 1.06)
3+	68	1161	1.22 (1.05, 1.42)
Parity (tenure prior to age 45)^b^			
Nulliparous	19	484	1.44 (0.83, 2.49)
1 to 2	50	896	0.95 (0.78, 1.17)
3+	58	848	1.39 (1.06, 1.81)

^a^All models are adjusted for age, overweight, education, parity and smoking history

^b^Models for tenure prior to age 45 are restricted to participants age 45 or over

## Discussion

We have conducted a large and comprehensive study characterizing cancer rates among U.S. cabin crew relative to the general U.S. population, which adds to the relatively sparse literature regarding cabin crew health and has included profiling a wide range of cancers. Consistent with previous studies reporting on cancer incidence and mortality among flight attendants, we report a higher prevalence of breast, melanoma and non-melanoma skin cancers (comprising basal cell and squamous cell carcinomas) among this occupational group relative to the general population. This is striking given the low rates of overweight and smoking among flight attendants in our study population, which we take to be indicators of general health and healthy behaviors, as well as being independent risk factors for some cancers [1, 11]. We also report associations between job tenure as a flight attendant and several cancer outcomes, consistent with previous U.S. and European studies [7–11], though we note that our reliance on cancer prevalence rather than incidence complicates the interpretation of our findings with regard to the timing of both work exposures and cancer outcomes, and the conflation of cancer incidence and survivorship. Nevertheless, our study extends the sparse literature on this important topic, confirms previous findings, and is the first study to note an increase in non-melanoma skin cancer among U.S. cabin crew (consistent with studies of European cabin crew and pilots). Our work informs future research directions regarding the health of this understudied group of workers and highlights the question of what can be done to minimize the adverse exposures and cancers common among cabin crew.

Our finding of a greater prevalence of breast and skin cancers among flight attendants is consistent with most of the epidemiologic literature on this topic to date [7–11]. As noted above, our study is the first to show an increase in non-melanoma skin cancer among U.S. cabin crew relative to the general population, which replicates findings among European flight attendants and pilots [11, 24]. We also observed that job tenure as a flight attendant was associated with the prevalence of non-melanoma skin cancer, as well as breast cancer (within parity subgroups), among females. We were also able to conduct SPR analyses among crew with in-flight secondhand smoke exposure prior to 1988 and found that some associations were strengthened among this subset of participants. Interpretation of the latter results is somewhat hampered by the fact that participants’ occupational secondhand exposures ended by 1998 at the latest, and studies regarding smoking or secondhand smoking exposures and breast and skin cancers have reported mixed results [25, 26]. However, secondhand smoke has been linked to breast and skin cancers in some studies and is certainly a potential risk factor for these cancers, and unlike for cardiovascular disease, smoking-related risk of cancer never falls to baseline, even years after cessation of the exposure [25, 26].

Our results are also consistent with cabin crews’ occupational exposures to ionizing radiation [2, 6], Circadian rhythm disruption [3], historical exposures to in-flight secondhand smoke [5], and ongoing exposures to other chemical agents [2], most of which are classified as confirmed or probable carcinogens in humans [27–29]. Ionizing radiation is a known causal factor for non-melanoma skin cancer and breast cancer [27], whereas the studies regarding melanoma in relation to ionizing radiation are more conflicted [30]. It should be noted that cabin crew have the largest annual ionizing radiation dose of all U.S. workers (e.g. 3.07 mSv vs. 0.59 mSv for U.S. Department of Energy workers) [5]. These exposures can easily exceed guidelines released by the NCRP or the International Commission on Radiological Protection [6, 31]. Although we evaluated job tenure prior to age 45 or age 40 in relation to cancer prevalence, in part to isolate the potential effects of ionizing radiation exposure at younger ages, these restrictions generally did not meaningfully alter our results. This may be because ionizing radiation exposure is also important to cancer risk at older ages, and because it is difficult to disentangle the relevant exposure years in our study population, which has a median tenure of 19 years of employment and for which cancer diagnosis date was not recorded. One possible exception is for breast cancer, for which associations were somewhat stronger when evaluating tenure prior age 45 rather than lifetime tenure. These results, while imprecise and requiring replication in a study that estimates cosmic ionizing radiation exposure directly (rather than using tenure as a proxy), may suggest that flight-related exposures are most important to breast cancer risk when occurring at earlier ages.

We report associations between duration of employment as a flight attendant and breast cancer risk among women who had three or more children, with some evidence of an association among nulliparous women as well, though the latter association was imprecise. Nulliparity is a risk factor for breast cancer, and women who are parous may be less susceptible to the effects of carcinogenic exposures on the breast due to breast cell differentiation occurring after a first pregnancy [32, 33]. Hence, our findings of a somewhat stronger association between job tenure and breast cancer among nulliparous women is consistent with the current state of biologic and epidemiologic knowledge, though it should be noted that the few relevant previous studies among cabin crew did not show increased risk of breast cancer among nulliparous relative to parous participants [20, 21, 34]. Our findings of a stronger association between tenure as a flight attendant and breast cancer among women with three or more children is, interestingly, consistent with two other recent publications on this topic among cabin crew [20, 21]. The authors of the latter studies, which evaluated breast cancer in relation to calculated cosmic radiation exposure and Circadian rhythm disruption, hypothesized that these unexpected results may be due to Circadian rhythm changes from shift work and crossing time zones [21], especially since flight attendants report a much higher rate of sleep disorders and disturbances relative to the general population [11, 12] and these effects may be exacerbated among women with young children who have greater sleep disruptions from both their home and work lives [21].

Limitations of our study include its cross-sectional design, which precludes inferences about causality, as an observed association may reflect the effect of flight attendant work on a given condition, or the effect of an outcome on a factor related to employment as a flight attendant. Use of structured questionnaires, as in our study, aims to minimize this bias. We also note a further limitation that the date of cancer diagnosis was not recorded in the FAHS questionnaire. Hence, some reported cancers may have been diagnosed prior to employment as a flight attendant, and some flight attendant work (i.e. exposure) may have occurred following a cancer diagnosis, making the direction of the potential bias unclear. These limitations are counteracted in part by our analyses evaluating job tenure prior to age 40 and 45 years in relation to cancer prevalences, as many cancers, including of the skin and breast, occur later in life. Therefore, this restriction increases the probability that the exposure of interest occurred prior to the reported cancer outcome.

Another potential limitation of our study involves the question of whether a population of flight attendants is sufficiently comparable to the general U.S. population with regard to cancer risk factors, and whether differences in risk factors may introduce bias to the SPRs. For example, we report substantial differences in racial profile, smoking status, overweight prevalence, and number of live births between the FAHS and NHANES cohorts, all of which are related to the risk of various cancers. We have counteracted this issue in part by restricting the NHANES comparison group to currently employed adults with at least a high school degree and above a certain income to poverty ratio, and by conducting sensitivity analyses restricting to non-Hispanic white participants that showed no meaningful differences from our main results. We also note that the FAHS includes a substantially smaller percentage of current smokers and overweight participants than the NHANES population, which would be expected to decrease the risk of several cancers, whereas we consistently observed increased cancer SPRs. At the same time, we should note that FAHS participants had fewer children than NHANES participants (which elevates the risk of breast cancer), though this is in part ameliorated by the fact that we observed associations between tenure as a flight attendant and breast cancer within parity subgroups. Even with the above reported sensitivity analyses, we acknowledge that the potential for residual confounding by cancer risk factor profile differences between the two study populations (such as for race and parity) still exists.

In addition, health outcomes in our study and in the NHANES were based on self-report; validation through medical records was not possible due to the scope and cost of this endeavor. Validity of self-reported health outcomes varies by study population and the outcome of interest. Sensitivity and specificity of self-reported outcomes relative to medical records or linkage to disease registries were found to be moderate to high for common cancers (including breast cancer and melanoma), particularly among those with higher socioeconomic status, such as in our well-educated cohort [35]. However, this has not been the case for non-melanoma skin cancer. We should note that non-melanoma skin cancers are excluded from most U.S. cancer registries and may be under-reported by those that do include it [36]. This may explain why non-melanoma skin cancer assessed through the California Cancer Registry was not related to flight attendant work in a previous study [9], in contrast to many other studies conducted among cabin crew and pilots, including our own research presented here [11, 24].

A further limitation of our study is that we recruited flight attendants from a mix of company rosters, on-site airport recruitment, and an online/social media presence. Recruiting volunteer participants not recruited from employee files may have contributed to selection bias. For example, volunteer participants may differ from those recruited using a more randomized approach in terms of various factors, including their socioeconomic status, attitude toward health research, and factors related to time and ability to complete online surveys (which may also be related to health), as discussed in a recent analysis with regard to online recruitment in the Heart eHealth Study relative to NHANES [37]. However, the above analysis reported that, while selection bias was likely on a variety of factors, such as gender and marital status, it was much less likely to affect internal (rather than external) validity of exposure-outcome associations [37]. This is likely to be especially true in a relatively homogenous workforce than in a general population study recruited online. It is also important to note that an online recruitment strategy has many advantages in terms of efficiency, reliability of data collection and coding, and the ability to reach a wider range of potential study participants [37].

Our study may have attracted a disproportionate number of flight attendants with cancer, leading to detection bias, as flight attendants with worse health are likely to be more motivated to participate in an epidemiological study of flight attendant health, are likely to attend regular medical check-ups (this is true for flight attendants in general), and the question of cancer risk in relation to flight exposures is well known within the aviation community. However, it is reassuring that our results are consistent with previous studies that recruited participants from employee rosters [8–10].

Additional limitations of our study include reliance on job tenure as a surrogate for occupational exposures, lack of correction for multiple testing, insufficient power to evaluate less common cancers, and insufficient information on confounders for some cancers. In particular, we were not able to control for leisure-time UV exposure when evaluating skin cancer risk, though it should be noted that a large study found no difference in sunbathing habits between flight attendants and the general population [38]. We plan to evaluate specific exposures in future individual exposure-outcome analyses. Finally, we note that our reliance on prevalence rather than incidence of cancer confuses the issues of cancer risk and survivorship in interpreting our results. This is in part ameliorated by the fact that breast and skin cancers have relatively low mortality rates (especially for basal cell carcinoma, which is not considered fatal or disabling), and that we are comparing to prevalence rates in NHANES as well. Nevertheless, the limitation remains, and it is also important to note that flight attendants may differ from the general population of U.S. workers with regard to health insurance access, paid leave policies, and other benefits that could affect survivorship, and may be more likely to have access to an urban center with better quality health care for cancer treatment. It is reassuring that our results are consistent with previous studies that relied on cancer incidence [9, 10].

Strengths of our study include access to the resources of a large cohort of cabin crew with information on a range of cancer outcomes, work experiences, and potential confounders. In addition, online questionnaires are an increasingly popular option in epidemiologic research, including high profile studies such as the Millennium Cohort and the Nurses’ Health Study 3 [39]. This mode of data collection allows for validation checks, reduced data entry and coding errors, personalized question administration, convenience to participants, equal or better validity compared to hard copy questionnaires, and the collection of metadata, such as date, time, and time to completion, which can be used for quality control and sensitivity analyses [39].

Our study findings contribute to the sparse literature on flight attendant health, which may also be applicable to passengers, especially frequent flyers. Conducting high quality studies within this group of workers is important given that U.S. cabin crew are subject to fewer protections than most workers in this country and relative to flight attendants working in the European Union (EU). For example, the EU requires airlines to monitor radiation dose, organize schedules to reduce radiation exposure, and inform workers of current studies [40].

## Conclusions

We have conducted a large and comprehensive study characterizing cancer prevalence rates among flight attendants relative to the general population. Despite low smoking and obesity levels indicative of positive health behaviors, we report that flight attendants have elevated rates of several cancers, especially breast, melanoma, and non-melanoma skin cancers. These results are consistent with previous findings regarding flight crew health. Ours is the first study to report an elevated rate of non-melanoma skin cancer in a U.S. flight attendant cohort (consistent with European studies). Some of these cancers were also related to tenure as a flight attendant, overall or within subgroups of parity in the case of breast cancer. Our results yield information to guide future research regarding the health of this understudied group of workers, which can also be considered when evaluating how to improve health and quality of life among cabin crew.

## Additional files

Additional file 1: **Table S1.** Participant Characteristics, Harvard Flight Attendant Health Study (2014–2015) and NHANES (2013–2014). (XLS 13 kb)

Additional file 2: **Table S2.** Comparative age-adjusted cancer prevalence in the Harvard Flight Attendant Health Study (FAHS, 2014–2015) and NHANES (2013–2014), evaluating only flight attendants with occupational smoking exposure prior to the year 1988. (XLS 10 kb)

## Abbreviations

CI: Confidence interval
EU: European Union
FAHS: Flight attendant health study
NCRP: National Council on Radiation Protection
NHANES: National Health and Nutrition Examination Survey
OR: Odds ratio
SPR: Standardized prevalence ratio
